# Computer‐Assisted Performance‐Based Assessment for Mental Health: A Scoping Review

**DOI:** 10.1002/pchj.70086

**Published:** 2026-02-27

**Authors:** Hanya Li, Shuang Li

**Affiliations:** ^1^ School of Educational Technology, Faculty of Education Beijing Normal University Beijing People's Republic of China

**Keywords:** assessment scenario, computer‐assisted technology, machine learning, mental health, performance‐based assessment

## Abstract

Adolescent mental health is foundational to personal development, yet it faces escalating challenges globally. While traditional assessment methods lack objectivity and ecological validity, integrating computer‐assisted technology (CAT) into performance‐based assessments (PBAs) offers a promising pathway. This review, following the PRISMA‐ScR reporting standard, analyzed 89 articles (2015–2025) to map the assessed components, CAT applications, and scenario diversity in mental health PBAs. Analysis revealed a research emphasis on mental disorders, with critical domains for adolescent development remaining significantly understudied. CATs significantly enhanced PBAs through data analysis, data acquisition, scenario creation, and tool digitization. PBA scenarios are diverse, demonstrating the adaptability of PBAs for multidimensional mental health assessment. Prioritizing the design of PBAs for social–emotional and adaptive assessment is critical for the early identification of adolescent mental health issues. Furthermore, advancing predictive analytics and leveraging large language models for feedback generation are promising ways to unlock CAT's potential in enhancing PBAs. Importantly, integrating and adapting scenarios from validated scales by CATs into PBAs could further enhance assessment typicality and reliability.

## 1 Introduction

Mental health is a critical foundation of youth development, supporting independence, academic achievement, and the formation of lifelong relationships (Patton et al. 2016). Recent data highlight the growing prevalence of mental health challenges among adolescents, with 15% of adolescents aged 10–19 years worldwide experiencing mental disorders in 2021 (IHME 2021). In the United States, 40% of high school students reported persistent sadness or helplessness in 2023 (Centers for Disease Control and Prevention 2024). These widespread issues not only profoundly impact individuals but also place significant burdens on health care and education systems, indicating an urgent need for more effective mental assessments and intervention strategies.

Effective assessment begins with a clear and comprehensive conceptualization. Mental health definitions range from the absence of diagnosable disorders to an emphasis on general well‐being (Wren‐Lewis and Alexandrova 2021). Different perspectives directly shape the scope and focus of mental health assessments, thus influencing the constructs measured. Traditional methods, such as self‐reports and clinical checklists, are favored for their convenience and cost‐effectiveness. However, social desirability bias and inaccurate self‐perception limit their insights (Feldon et al. 2015). They also lack ecological validity, failing to capture real‐world behaviors and unconscious psychological states. This limitation is particularly problematic in measuring adolescent mental health, which is unstable (Patel et al. 2023).

Performance‐based assessment (PBA) offers a promising alternative to traditional methods by evaluating observable behaviors in realistic or simulated scenarios. It effectively detects subtle impairments frequently overlooked by traditional methods, such as cognitive deficits and social skills (Angers et al. 2024; Keleman et al. 2024). Tasks such as the Multiple Errands Test (MET) (Shallice and Burgess 1991) and the UCSD Performance‐Based Skills Assessment (UPSA) (Patterson et al. 2001) show strong ecological validity, linking assessments with real‐life challenges (Dawson et al. 2009; Green et al. 2015). Moreover, embedding assessments in interactive scenarios enhances their accuracy and participant engagement (Aubrey 2011; Suastra and Menggo 2020). For example, the Shape School task (Espy 1997) is an accessible and enjoyable assessment for preschoolers (O'Meagher et al. 2019; Power et al. 2023).

The integration of computer‐assisted technology (CAT), such as virtual reality (VR) and machine learning (ML), further expands the potential of PBA (Al‐Ansi et al. 2023; Fazil et al. 2024). CATs enable scalable, objective, and ecologically valid mental health assessments by simulating naturalistic scenarios and processing complex data (Cerasuolo et al. 2024). Despite significant advancements, the state of computer‐assisted mental health PBA remains insufficiently understood, leaving key questions unanswered. Specifically, what mental health components are measured through computer‐assisted PBAs? What PBA scenarios are employed? What techniques are applied, and how do they facilitate mental health PBAs? To address these gaps, this study systematically synthesizes existing research on computer‐assisted mental health PBAs, focusing on their conceptual frameworks of mental health, the diversity of assessment scenarios, and the role of CAT in measuring various mental health dimensions. Through a comprehensive and structured analysis, this research not only deepens the understanding of computer‐assisted mental health PBAs but also offers actionable recommendations to advance the precision and applicability of mental health assessments.

### 1.1 Divergent Understandings of Mental Health

Mental health is a complex and multidimensional construct that varies across cultures and contexts. Traditionally, it has been narrowly defined as the absence of diagnosable mental disorders, as reflected in psychiatric frameworks such as the *DSM‐5*, which classifies over 300 conditions (APA 2013). While this framework aids in clinical diagnosis and treatment, it has been criticized for privileging pathology and overlooking broader dimensions of mental health as a dynamic and positive construct (Li et al. 2024; Murphy 2017).

In contrast, holistic perspectives—often grounded in positive psychology—define mental health as the ability to adapt and self‐manage under stress, emphasizing psychological capacities necessary for thriving, such as emotional stability, meaning, resilience, and positive relationships (Huber et al. 2011; Huppert and So 2013; Seligman and Csikszentmihalyi 2000). It aligns with definitions of the American Psychological Association (APA 2018) and the World Health Organization (WHO 2022), which frame mental health as well‐being. However, the overlap between terms such as mental health, well‐being, and mental well‐being remains contested (Keyes 2013). While positive mental health can contribute to flourishing, conflating it with general well‐being risks imposing unrealistic standards and oppressive expectations (Keller 2020; Wren‐Lewis and Alexandrova 2021).

These divergent understandings have shaped the development of varied mental health measurement tools, each corresponding to specific definitions and components. Clarifying the conceptual foundations is thus essential for developing precise and effective assessments, particularly for adolescents whose mental health is shaped by complex developmental factors. This need underpins our effort to investigate the components commonly assessed by PBAs and the tools employed across different dimensions.

### 1.2 CATs In Mental Health Assessment

CATs have enhanced the efficiency and flexibility of mental health assessment by digitizing traditional PBA tools and creating assessment scenarios. For example, Moore et al. (2015) developed a mobile version of the UPSA that achieved 87% accuracy in identifying schizophrenia patients. Unlike traditional PBAs, which rely on resource‐intensive in‐person observations (Greenwood et al. 2016), CATs like VR can create controlled and immersive environments that allow researchers to elicit cognitive and behavioral responses with greater precision (Bell et al. 2020). For example, the VR adaptation of the MET automates data collection in a virtual supermarket, reducing implementation costs while increasing task complexity and flexibility (Rand et al. 2009).

Beyond convenience, CATs enable the collection of multidimensional data that goes far beyond traditional item responses. Process data such as keystrokes, eye‐tracking patterns, and action sequences offer deeper insights into psychological states (Jiao et al. 2021). When combined with physiological and behavioral measures, VR paradigms have been shown to be particularly valuable in assessing some mental disorders (Wiebe et al. 2023). These high‐dimensional datasets also facilitate advanced analytic methods, such as clustering or word embedding, to extract meaningful insights (Qorich and El Ouazzani 2024).

Furthermore, CATs enhance objectivity by mitigating biases in human observation and scoring. Modern computer systems can detect subtle behavioral and emotional cues, such as microexpressions, thereby improving the reliability of assessments (Eileen Brown 2017). ML and large language models (LLMs) further extend this capacity, offering advanced pattern recognition and predictive accuracy (Hodges 2021). For example, Lau et al. (2023) demonstrated the effectiveness of fine‐tuned LLMs in detecting depression through parameter‐efficient tuning techniques.

Despite these advances, insufficient interdisciplinary collaboration, technical challenges in implementation, and the lack of validation hinder the broader adoption of CATs in mental health PBAs. To date, applications of CATs in PBAs across diverse mental health dimensions have not been comprehensively mapped. Bridging these gaps is essential to fully realizing the potential of CATs in advancing mental health research and assessment practices.

### 1.3 Definition of Key Concepts

#### 1.3.1 Mental Health

As discussed above, existing definitions of mental health range from a pathology‐centered view that equates it with the absence of disorders to a more idealized notion overlapping with general well‐being. This study adopts a pragmatic and balanced definition: mental health is the capacity for psychological stability and adaptability when facing life challenges. It is a fluid condition influenced by individual and environmental factors (WHO and UNICEF 2024).

Informed by major mental health frameworks (APA, 2013; Keyes 2002; Ryff and Keyes 1995; WHO 2022), maintaining this balance requires (a) the absence of severe mental disorders, (b) basic cognitive and social skills integral to daily life (Moritz et al. 1995), (c) effective emotional regulation (Brandão et al. 2023), (d) adaptability to unpredictable events (Klanker et al. 2013), and (e) appropriate self‐cognition (Henriksen et al. 2017; Koban et al. 2021). Crucially, societal barriers must be distinguished from individual limitations because they may impede functioning without signifying poor mental health (Herrman 2016). This working perspective provides a robust framework for assessing and fostering mental health, particularly in adolescents, by equipping them with the necessary abilities and skills to navigate the challenges in their developmental journey.

#### 1.3.2 Performance‐Based Assessment

In education, PBAs involve structured tasks that simulate authentic challenges, allowing individuals to demonstrate their learning processes while generating tangible outcomes (Adri et al. 2022). They not only measure cognitive mastery but also provide feedback on practice (Suastra and Menggo 2020). In the context of mental health, PBAs refer to the use of simulated or real‐world tasks to evaluate individuals' mental states. This method provides an effective framework for mental health measurement and feedback, linking abstract mental concepts with tangible performance.

#### 1.3.3 Computer‐Assisted Technology

In the field of psychometrics, CATs broadly refer to a range of computerized tools and systems used throughout the assessment process, including hardware (e.g., input/output devices, sensors, and VR/AR devices), software platforms (e.g., test administration and data management platforms), and algorithmic approaches (e.g., ML, natural language processing, and LLMs). The core purpose of CATs is to assist assessment delivery, response capture, scoring, data processing, and result interpretation through automation, standardization, and enhanced interactivity (AERA et al. 2014; Bauer 2017).

#### 1.3.4 Performance‐Based Assessment Scenario

In the context of computer‐assisted PBA, a scenario refers to a temporal sequence of contextual elements in which actions and events unfold and can be systematically observed (Ulbrich et al. 2015). Scenarios may involve structured experimental tasks or naturally occurring behaviors, provided they elicit measurable performance that reflects underlying psychological constructs. By embedding assessment tasks in structured scenarios, PBAs provide ecologically valid conditions that allow for direct or indirect observation and multimodal data capture.

### 1.4 Previous Reviews

Several reviews have examined advanced technologies and PBAs in mental health, focusing on cognitive functions, specific populations, and technological innovations (see Table 1). For example, Raimo et al. (2024) conducted a meta‐analysis showing that visuospatial and language abilities are strongly correlated with daily living activities, particularly when evaluated with PBAs. Similarly, Gawęda et al. (2024) demonstrated that PBAs are effective in identifying cognitive biases in schizophrenia spectrum psychoses. Iceta et al. (2021) reviewed neurocognitive function PBAs in individuals with binge eating disorder or food addiction. Population‐specific research further underscores the versatility of PBAs. For example, Su et al. (2023) highlighted the adaptability of PBA tools for preschool‐aged children across different cultural and developmental contexts. Collectively, these studies demonstrate the ability of PBAs to detect subtle impairments and address diverse clinical needs.

**TABLE 1:** Summary of related reviews (arranged by year).

Author (year)	Approach	Time period	Included articles	Review focus
Wongkoblap et al. (2017)	Systematic review	2010–2017	48	The scope and limits of applying cutting‐edge techniques to generate predictive analytics for mental disorders.
Su et al. (2020)	Systematic review & meta‐analysis	2014–2019	57	The application of deep learning to the analysis of different types of data related to mental health conditions.
Iceta et al. (2021)	Systematic review & meta‐analysis	2010–2020	42	The cognitive dysfunctions or difficulties underpinning binge eating disorder and food addiction.
Kusuma et al. (2022)	Systematic review & meta‐analysis	2002–2021	35	The performance of ML models in predicting longitudinal outcomes of suicide‐related outcomes.
Su et al. (2023)	Systematic review	1989–2022	98	The current application of executive function PBA tools for Chinese preschoolers.
Cerasuolo et al. (2024)	Systematic review	2022–2023	20	The possibility of employing VR systems in the assessment and diagnosis of autism.
Gawęda et al. (2024)	Systematic review	1984–2023	308	The role of cognitive biases as assessed by PBAs for schizophrenia spectrum psychoses.
Raimo et al. (2024)	Meta‐analysis	1982–2022	194	The nature and strength of the association between cognitive functioning and daily living activities.

Other reviews have explored the integration of specific technologies in mental health assessment. Wongkoblap et al. (2017) synthesized work on social media data–based prediction of mental disorders, noting the dominance of text‐based approaches. Cerasuolo et al. (2024) highlighted the potential of VR in diagnosing autism spectrum disorder (ASD) by creating objective, ecological, and engaging scenarios. Building on this, Su et al. (2020) demonstrated the potential of deep learning (DL) for developing disease risk prediction models, while Kusuma et al. (2022) showed that ML approaches can outperform traditional models in predicting longitudinal suicide‐related outcomes, offering both scalability and predictive power.

Previous reviews have offered valuable insights into the application of CATs and PBAs in mental health assessment, but their scope has been fragmented. Most focus either on specific mental health or on particular technologies, which limits a comprehensive understanding of how CATs and PBAs map into the multidimensional nature of mental health. Moreover, little attention has been given to PBA scenarios, which are important to ensure that PBAs align with the constructs they aim to measure (Mutweleli et al. 2024). To address these gaps, the present scoping review systematically examines the interplay between CATs, the mental health constructs they assess, and the scenarios in which PBAs are implemented.

### 1.5 Research Rationale, Aims, and Questions of the Current Review

The current review differs from previous reviews in three aspects. First, unlike previous reviews that focused on isolated mental health constructs, this study adopts a multidimensional perspective, providing a comprehensive review of the research on and application of computer‐assisted mental health PBAs across cognitive, emotional, social, and other dimensions. Second, rather than emphasizing specific technologies, this review explores how CATs broadly enhance mental health PBAs. Third, this review examines different scenarios employed in PBAs, identifies how they align with different dimensions of mental health, and highlights the role of CATs in tailoring assessments to these scenarios. By integrating these considerations, this study seeks to develop a robust framework for understanding the application of CATs and PBAs in mental health assessment. Specifically, it aims to clarify which PBA scenarios are frequently used to assess specific mental health dimensions and how CATs can optimize assessments.

To achieve these objectives, this review poses the following research questions:

*What mental health components are typically measured via computer‐assisted PBAs*?

*What roles do CATs play in enhancing mental health PBAs*?

*What scenarios are used in computer‐assisted PBAs for mental health*?

## 2 Method

This study presents a scoping review of the use of CATs in evaluating mental health through PBAs. The approach taken for this scoping review was informed by the Preferred Reporting Items for Systematic reviews and Meta‐Analyses extension for Scoping Reviews (PRISMA‐ScR; Tricco et al. 2018).

### 2.1 Search Strategy

A comprehensive literature search was conducted across five databases: Web of Science, EI Engineering Village, Scopus, APA PsycINFO, and PubMed. Studies were searched from 2015 onward as this period marks the development of CATs and their integration into psychological assessment research. The first search was performed on September 13, 2024, using structured keyword combinations related to computer‐assisted mental health assessment. The terms were organized into three conceptual domains and combined with Boolean operators (see Table 2). To ensure sensitivity and coverage, the search strings were piloted and refined in several iterations until they consistently retrieved key benchmark studies. There were no age limits for participants so that the mapping of computer‐assisted PBAs for mental health could be as complete as possible, as this is a new and limited study topic. A second search was conducted on December 31, 2025, following the same process. The complete search strategies are provided in Appendix A.

**TABLE 2:** Search strategy to identify articles on computer‐assisted mental health assessment.

Search focus description	Example terms
1. Identifies studies addressing mental health aspects	Mental health, well‐being, mental disorder…
2. Identifies studies involving the assessment of mental health conditions	Assessment, measure, test
3. Identifies studies using CATs in mental health assessment	Computer assisted, computerized, artificial intelligence, machine learning
Final logic	1 AND 2 AND 3

### 2.2 Inclusion and Exclusion Criteria

The titles and abstracts of the articles retrieved with the specified search terms (see Table 2) were screened according to the inclusion criteria. Only English‐language articles were considered. Further inclusion criteria are provided in Table 3; exclusion criteria are the inverse.

**TABLE 3:** Inclusion criteria.

Inclusion Criteria
1. Focuses on mental health or its components
2. Includes an assessment of mental health or its components
3. Performed in real or simulated scenarios
4. Incorporates CATs to aid assessment
5. Based on data and empirical observations
6. Evaluates mental health components at the individual level

### 2.3 Screening and Data Management

Search results from all databases were imported into reference management software, where duplicates were identified through standardized bibliographic information and removed. Study selection proceeded in two stages: (a) titles and abstracts were screened against the inclusion criteria, and (b) full texts of potentially eligible studies were reviewed for confirmation. Two reviewers conducted independent duplicate screenings of the title, abstract, and full text, achieving significant inter‐rater agreement (Cohen's *κ* = 0.83). We developed a standardized data tool to extract information on studies, including populations, assessment domains, applied CATs, and PBA scenarios. Two reviewers independently extracted data and resolved discrepancies through consensus. Appendix B provides the detailed information.

## 3 Results

### 3.1 Search Results

**FIGURE 1:**
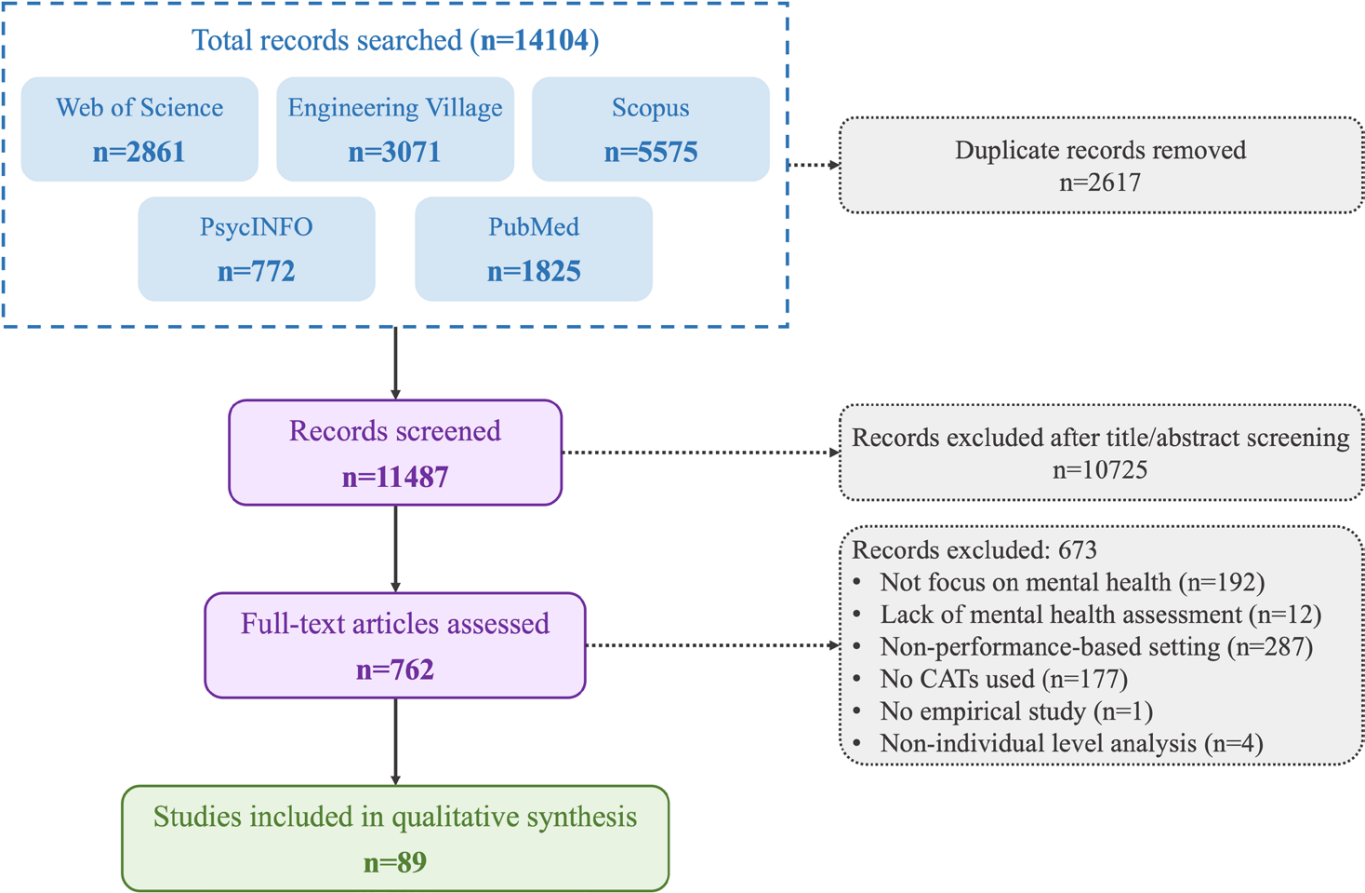
Flow diagram depicting the article screening process.

A total of 14,104 articles were retrieved across all databases. After the removal of duplicates (*n* = 2617), 11,487 articles were screened by title and abstract. The full texts of the remaining 762 articles were assessed, leaving 89 articles to be included in the review (see Appendix B, Figure 1 for screening flow).

**FIGURE 2:**
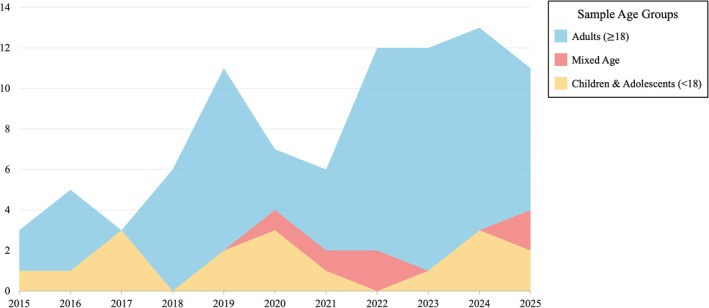
Distribution of screened articles by sample age group from 2015 to 2025.

The included studies were conducted across multiple countries, with the largest number of publications originating from the United States (26, 29%), China (13, 15%), and India (8, 9%). Validation studies accounted for most of the literature (57, 64%), followed by case–control studies (15, 17%), whereas longitudinal (4, 4%) and intervention‐based studies (2, 2%) were relatively rare. Publication volume showed an upward trend over time, particularly after 2017 (see Figure 2). Most studies focused on adult populations (48, 54%); only 17 articles examined children or adolescents exclusively. Sample sizes were generally modest, with over half of the studies involving fewer than 100 participants (51, 57%), whereas 19 studies included samples exceeding 200 participants.

### 3.2 Mental Health Components

**FIGURE 3:**
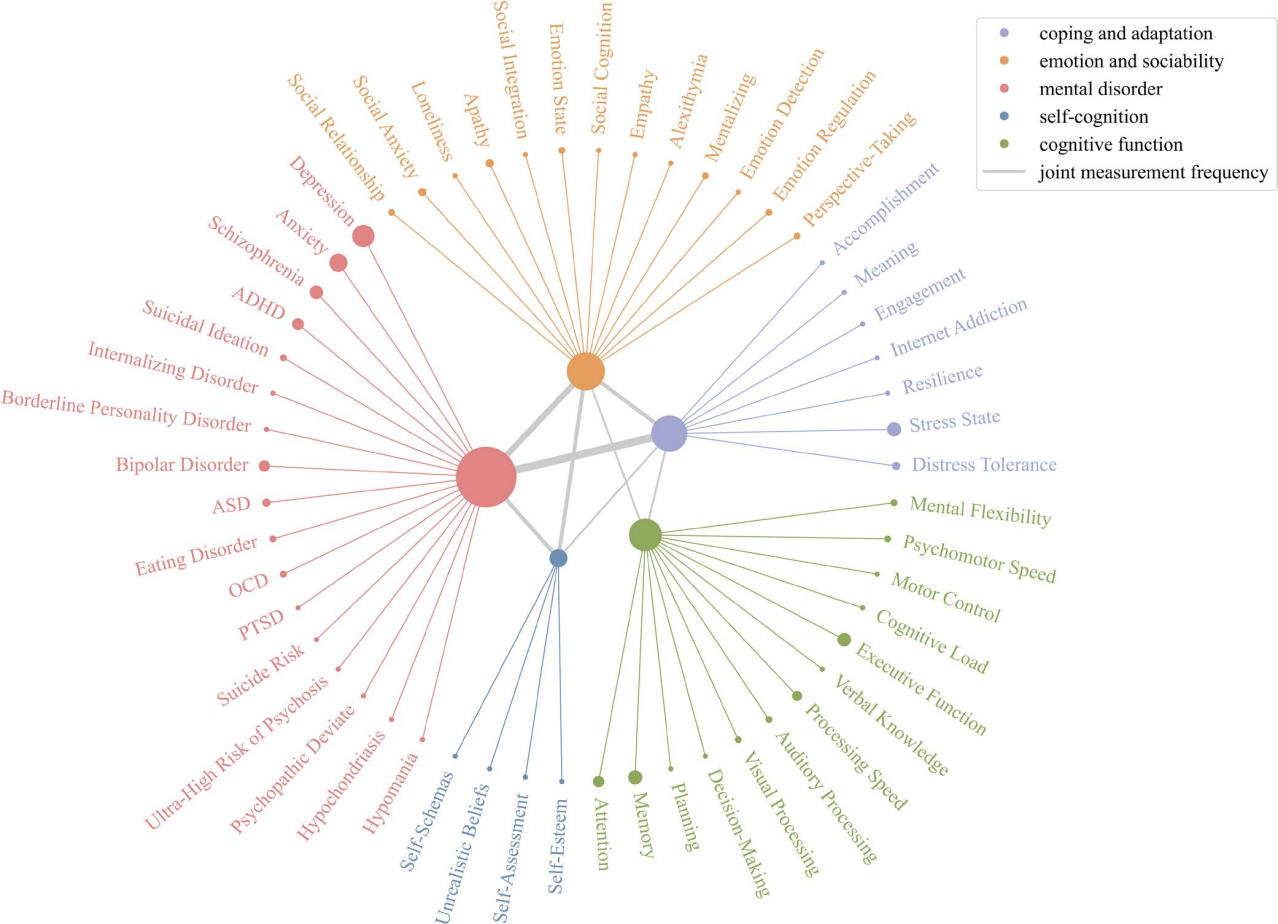
Network diagram of mental health components assessed in the reviewed articles. Node size reflects the number of studies assessing each category or component; gray line thickness represents the number of studies jointly measuring the two categories.

In line with the established definition of mental health, this review categorized the measured variables into five main categories: mental disorders, cognitive functions, emotional and social competence, coping and adaptation, and self‐cognition. Figure 3 presents the components included in these five categories and the number of articles measuring them. Mental disorder was the most frequently measured category (49 articles, including 41 exclusively focused on this domain). Self‐cognition was rarely assessed, with only four articles. Although most studies targeted a single category, 12 combined two or more domains—most often mental disorders with coping and adaptability.

#### 3.2.1 Mental Disorders

The reviewed studies assessed a range of mental disorders, such as depression, anxiety, schizophrenia, and bipolar disorder, with 8 studies focusing on adolescent populations. Overall, 26 studies investigated depression and 16 assessed anxiety, with 13 addressing both conditions concurrently. Other mental health issues were also represented, albeit to a lesser extent. Schizophrenia was assessed in nine articles, typically using patient‐control comparisons. Disorders with higher prevalence in specific age groups, such as attention‐deficit/hyperactivity disorder (ADHD) and ASD, were evaluated in only nine articles. Suicidality was explicitly assessed in three articles, including Liu, Zhong, et al. (2022) and Liu, Lu, et al. (2022), which recruited participants via a project's website and classified them into suicide risk classes based on preassessment interviews.

#### 3.2.2 Cognitive Functions

Cognitive functions, such as memory, attention, and decision‐making, enable individuals to acquire knowledge and carry out tasks. Cognitive impairments often coexist with mental disorders and can adversely affect daily functioning (McIntyre et al. 2013). This review identified 14 articles measuring cognitive functions through PBAs. The most articles, nine in total, focused on executive functions, with 4 evaluating memory and attention simultaneously. For example, Tezer et al. (2025) assessed the degree of cognitive impairment in children and adolescents with Juvenile Idiopathic Arthritis in terms of executive function, composite memory, working memory, complex attention, and 6 additional domains. Hoffmeister et al. (2025) assessed the elderly's memory, attention, executive functioning, and processing speed to detect early Alzheimer's disease.

#### 3.2.3 Emotion and Sociability

Emotion and sociability refer to individuals' abilities to recognize, regulate, and express emotions, as well as to initiate and maintain social relationships. This dimension captures factors essential for emotional stability and social functioning in both stressful and routine contexts. A total of 19 articles measured emotion and sociability, with 5 focusing on adolescent populations. For example, two articles evaluated adolescents' social perspective‐taking (Fantozzi et al. 2024; Pile et al. 2017), a core component of empathy that enables understanding others' thoughts and feelings.

In adults, emotion regulation was a key focus. Broch‐Due et al. (2018) compared emotion regulation skills between individuals with bipolar disorder and healthy controls, reporting significant differences. Emotion regulation is closely linked to emotion recognition (Rosenthal et al. 2011), which Myruski et al. (2019) assessed through facial morphing tasks. Regarding social anxiety, two studies examined it by requiring participants to deliver speeches in socially stressful settings (Harshit et al. 2024; Nowakowski et al. 2015).

#### 3.2.4 Coping and Adaptation

Coping and adaptation refer to the behavioral and cognitive strategies that individuals employ to manage stress, endure adversity, and adjust to life's challenges (Aspinwall 2004). Unlike emotion and sociability, which emphasize ongoing affective and interpersonal processes, coping and adaptation are situational responses to specific external challenges that emphasize goal‐directed strategies tailored to immediate demands (Marroquín et al. 2017).

Seventeen studies evaluated coping mechanisms and adaptation, including 10 that examined stress states across different populations. For example, Schoch‐Ruppen et al. (2018) assessed prenatal stress in pregnant women, emphasizing implications for both maternal well‐being and neonatal development, while Singh and Singh (2022) assessed stress in a broader cohort of 2500 Indian online users. Four studies focused on resilience and distress tolerance, such as Iasonidou et al. (2023), who examined women at varying risks of eating disorders under physical and cognitive stressors. Coping mechanisms were also linked to behavioral outcomes: Purwandari et al. (2020), for example, explored internet addiction in undergraduate students. Together, these findings underscore the importance of assessing coping strategies and their effectiveness in mitigating stress, enhancing resilience, and preventing maladaptive outcomes.

#### 3.2.5 Self‐Cognition

Self‐cognition refers to individuals' awareness and understanding of themselves, including their beliefs, perceptions, and assessments. It is conceptually and functionally distinct from cognitive functions, as impairments in these domains can be dissociated (Rutherford and Rogers 2003). Although studies examining self‐cognition represent a smaller subset of this review, they provide valuable insights. Ali et al. (2022) investigated unrealistic beliefs, that is, deviations from reality, that may shape negative self‐schemas, which are basic to one's self‐concept and can impact various aspects of mental health and behavior. McArthur et al. (2019) explored self‐schemas among adolescents, a developmentally sensitive group experiencing various changes.

### 3.3 Computer‐Assisted Approaches

**FIGURE 4:**
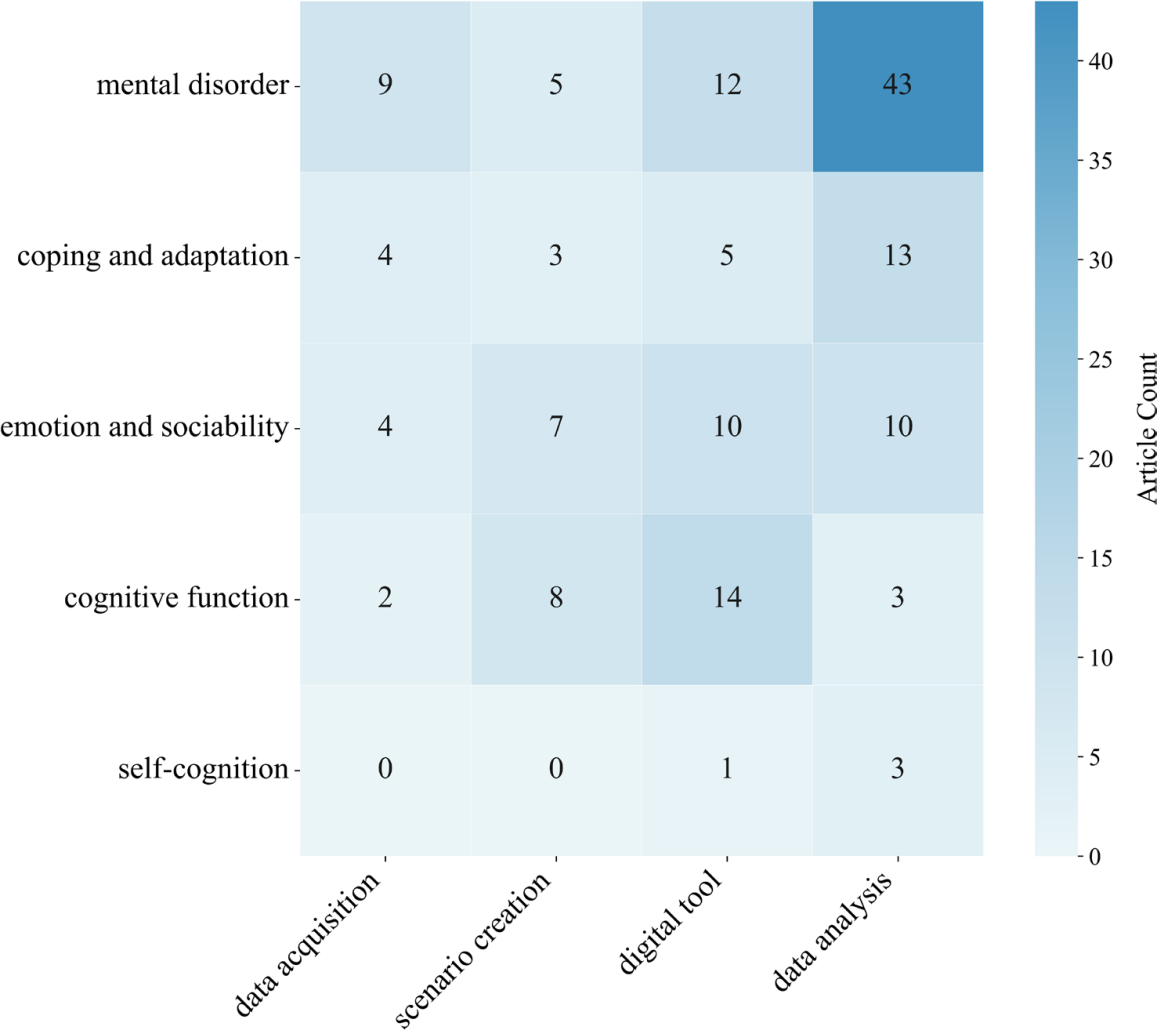
Applications of CATs across five mental health dimensions.

Based on the evidence‐centered design framework (Mislevy et al. 2003), this review classifies the implementation of CAT in PBAs into four types according to their roles in evidence generation and representation: tool digitization, scenario creation, data acquisition, and data analysis. Tool digitization converts traditional instruments into digital formats, enhancing standardization and procedural control through automated presentation and scoring. CATs like VR and AR strengthen assessments' ability to accurately elicit the targeted psychological processes by creating more immersive, realistic, and ecologically valid scenarios. Data acquisition broadens the range of observable evidence by collecting behavioral and physiological responses. Finally, data analysis, using statistical learning, ML, and DL models to extract and analyze evidence from multimodal data, improves predictive accuracy. Figure 4 shows their distribution across five mental health dimensions. Data analysis was the most prevalent application (60 articles), followed by tool digitization and scenario creation. Scenario creation was mainly used to assess cognitive function, emotion and sociability, while data acquisition and analysis were more often used for mental disorder PBAs. Tool digitization exhibited a more even distribution across cognitive function, emotion and sociability, and mental disorder domains.

#### 3.3.1 Data Analysis

Data analysis refers to using computational techniques to extract meaningful patterns from behavioral, textual, and physiological data. These methods fall broadly into four paradigms: feature engineering, architecture engineering, objective engineering, and prompt engineering (Liu et al. 2023).

Within our review, feature engineering was the most prevalent, applied in 57 articles. Grounded in domain expertise, it involves extracting key features (e.g., language use, facial expressions, and physiological reactions) to improve predictive accuracy and interpretability. For example, Purwandari et al. (2020) analyzed web browsing histories and selected five specific features to develop a classification model for internet addiction. Textual feature extraction methods such as LIWC (Pennebaker et al. 2007) and TF‐IDF (Salton and Buckley 1988) were frequently applied to social media posts or personal narratives. Schoch‐Ruppen et al. (2018) applied LIWC to analyze pregnant women's written texts, finding associations between prenatal stress and frequent use of negative‐emotion words.

Architecture engineering involves designing and selecting DL models. Roy et al. (2020) employed neural networks to create a binary model that detected suicidal ideation from tweets. Five articles combined feature and architecture engineering. Liu, Zhong, et al. (2022) and Liu, Lu, et al. (2022) compared the predictive performance of these two in text analysis for assessing mental state. Objective and prompt engineering were less frequently applied in mental health PBAs. Ding et al. (2025) extracted 159 audio features from speech tasks, developed a TCN‐MTA multi‐task model with personality‐assisted auxiliary tasks, and then refined it using a joint loss function to aid in early diagnosis of depression. Only one study employed prompt engineering, with Shin et al. (2024) applying fine‐tuned LLMs to detect depression through users' diaries. LLMs were also used as foundational models in the architecture engineering approach. Jiang et al. (2024) applied models such as DinoV2, WavLM, and LLAMA‐65B to multimodal interview data (audio, video, and transcripts). After encoding signals into high‐dimensional embeddings, multimodal fusion was employed to identify depression and anxiety.

#### 3.3.2 Physiological and Behavioral Data Acquisition

Physiological and behavioral data acquisition involves the use of CATs, such as the Internet of Things (IoT), eye‐tracking systems, and neuroimaging technologies, to capture real‐time and high‐resolution indicators of mental states. These technologies allow for precise measurement of neural activity, visual attention, and movement patterns, offering an objective basis for mental health PBAs.

In this review, 18 studies employed CATs to enhance data acquisition. IoT sensors, employed in six studies, enabled real‐time collection of physiological and behavioral data, capturing physiological states and movement patterns. Eye‐tracking technologies, reported in five studies, were used to assess visual attention and cognitive processing. By monitoring eye movements and fixations, researchers gained insights into information processing (Kim et al. 2022), attention allocation (Fantozzi et al. 2024), and emotional responses to visual stimuli (Broch‐Due et al. 2018; Jyotsna et al. 2023; Xiang et al. 2024). Electroencephalography (EEG) was used in five studies to capture electrical brain activity, allowing researchers to examine brain changes linked to ADHD (Öztoprak et al. 2017), anxiety (Yadawad et al. 2024), stress (Rajendran et al. 2024), depression (Stone et al. 2025), and emotional state (Raina 2025).

#### 3.3.3 Scenario Creation

Scenario creation involves designing immersive and interactive PBA scenarios that simulate real‐world or imaginary situations. These scenarios, often powered by VR and interactive game design, enable researchers to observe participants' behaviors and responses in ecologically valid contexts while maintaining experimental control.

In this review, 22 studies applied CATs to develop scenarios. Seven created VR scenarios, with settings ranging from imaginative (e.g., a spaceship; Marín‐Morales et al. 2021) to everyday environments, such as a supermarket (Tsai et al. 2021), kiosk (Kim et al. 2022), orchard (Danousis and Goumopoulos 2023), social scenarios (Kjærstad et al. 2022), and classrooms (Neguț et al. 2017; Parsons and Carlew 2016). These examples illustrate VR's versatility in constructing dynamic and context‐specific scenarios tailored to diverse research objectives.

Beyond VR, three studies adapted classic cognitive tests and incorporated them into video games. For example, Ibrahim et al. (2023) adapted five classic tests into a puzzle adventure game set in a village, where participants need to complete tasks like house renovation to evaluate their multiple cognitive functions. Additionally, the director task was used in two studies to simulate social communication under asymmetric information, assessing participants' capacities to infer others' mental states. Similarly, the Movie for Assessment of Social Cognition (MASC), adopted in two studies, immersed participants in a film‐based scenario where they answered questions about characters' mental states (Corsi et al. 2021; Poznyak et al. 2019). These methods combine narrative richness with standardized assessment, offering unique insights into social cognition.

#### 3.3.4 Digital Assessment Tools

In this review, 40 articles employed multiple computerized PBA instruments across various mental health dimensions. Several classic paradigms have been digitized. The stop‐signal task (SST), traditionally used to assess inhibition control (Morein‐Zamir and Sahakian 2010), was employed by Leontyev et al. (2019), who compared the predictive power of its standard and ML‐enhanced versions. The latter, which integrated mouse‐tracking data, demonstrated higher accuracy in assessing ADHD symptoms. Similarly, the Stroop task (ST), designed to measure conflicting information processing (Stroop 1935), appeared in nine studies targeting mental disorders, cognitive functions, and adaptation. Badr et al. (2023) used the ST to induce and evaluate stress, while Lu et al. (2021) employed a word‐face version to assess emotional conflict resolution skills.

### 3.4 PBA Scenarios

**FIGURE 5:**
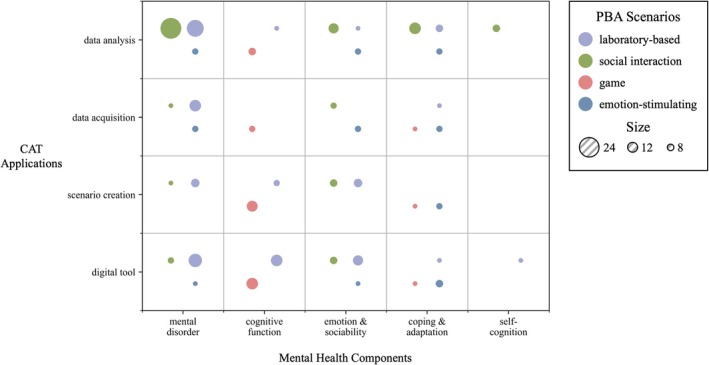
PBA scenarios assessing mental health under different CAT applications.

The PBAs reviewed encompass various scenarios, classified inductively according to their response‐elicitation mechanism, including laboratory‐based, social interaction, game, and emotion‐stimulating scenarios. Laboratory‐based scenarios were the most prevalent (39 studies), followed by social interaction scenarios (38 articles). Emotion‐stimulating scenarios appeared in 10 articles, while game scenarios were the least common (eight studies). As shown in Figure 5, laboratory‐based scenarios were employed across all mental health categories. Social interaction scenarios mainly focused on assessing mental disorders, with 27 articles enhancing data analysis through CATs. Game scenarios were applied primarily to measure cognitive functions, whereas emotion‐stimulating scenarios were commonly used to evaluate coping and adaptive abilities.

#### 3.4.1 Laboratory‐Based Scenarios

Laboratory‐based scenarios are highly controlled and standardized, designed to elicit specific psychological or behavioral responses using abstract or symbolic stimuli and tasks. Among these articles, 22 focused on mental disorders. Digitalized assessment tools were the most common CAT application in these scenarios, appearing in 26 articles.

Task accuracy and response time were widely used indicators, reported in 24 articles. Accuracy directly reflects task execution precision, while response time indicates information processing efficiency. Beyond these structured responses, several studies have expanded data acquisition by including multimodal features such as audio, video, and behavioral recordings to evaluate mental disorders. For example, reading tasks and the verbal fluency tasks facilitated audio‐based analysis (Bruno et al. 2023; Huang et al. 2023; Luo et al. 2024). Stark et al. (2022) quantified walking and balance performance using IoT, illustrating how behavioral data can enhance laboratory‐based PBAs.

#### 3.4.2 Social Interaction Scenarios

Social interaction scenarios rely on individuals' interaction with real people or virtual agents in socially meaningful contexts, such as role play, public speaking, teamwork, interviews, and social media engagement, to assess mental health through dynamic interpersonal behaviors. These scenarios were widely applied to measure mental disorders and emotion and sociability, with CATs typically enhancing data analysis.

Within these studies, social media analysis was especially prominent. Platforms like Facebook, Twitter, and Weibo provided data for assessing mental disorders, with features extracted from posts using CATs like LIWC and TF‐IDF. Researchers also analyzed additional parameters, such as browsing histories (Purwandari et al. 2020), comment threads (Hemtanon et al. 2020), follower counts, and posting time (Sivak and Smirnov 2020). Nguyen et al. (2018) conducted a longitudinal survey to predict depression and anxiety based on images that participants posted on social media platforms.

Interviews were another common method, reported in 10 articles. To predict suicidal ideation, Liu, Zhong, et al. (2022) and Liu, Lu, et al. (2022) analyzed eye contact in video data, while Wright‐Berryman et al. (2023) transcribed audio recordings for textual analysis. Simulated social pressure tasks, such as public speaking, mock job interviews, and simulated auditions, allowed researchers to observe stress‐related behaviors (Nowakowski et al. 2015; Oesten et al. 2023; Harshit et al. 2024; Yadawad et al. 2024; Kho et al. 2025). In addition, more naturalistic settings, such as teamwork projects in real workshops, were used to assess coping and adaptive abilities (Zeulner et al. 2024). Together, these approaches demonstrate the versatility of social interaction scenarios in capturing complex psychological processes across both structured and naturalistic contexts.

#### 3.4.3 Game Scenarios

Game scenarios embed PBA tasks within gamified and immersive environments, incorporating structured rules, goals, role assignments, rewards, and penalties. These scenarios all focus on evaluating cognitive functions, with CATs facilitating scenario creation and data analysis. Eight articles employed game scenarios, using performance metrics such as completion time, in‐game achievements, and behavioral responses as indicators of mental health states. VR games were designed to simulate real‐world environments, such as supermarkets and kiosks, to evaluate mild cognitive impairments and cognitive load (Kim et al. 2022; Tsai et al. 2021). Through CAT, eye‐tracking and sensor‐based data were frequently used to capture detailed behavioral responses. For example, Danousis and Goumopoulos (2023) employed sensors to capture the body positions and gestures of elderly participants in a VR fruit‐harvesting game, gaining insights into physical and cognitive engagement. Puzzle adventure games focus on structured narratives and still improve the immersion of the assessment without VR technologies (Ibrahim et al. 2023).

#### 3.4.4 Emotion‐Stimulating Scenarios

Emotion‐stimulating scenarios rely on affective cues to induce affective perturbation—shifting the individual from a neutral state to specific emotional arousal levels through passive or active induction. In reviewed articles, these scenarios are primarily used to examine coping and adaptation under emotion influence. CATs were predominantly applied in these studies for data acquisition and tool digitalization.

A total of 10 studies employed emotion‐stimulating scenarios, with five measuring coping and adaptive competencies. For example, frustration‐inducing tasks, such as the mirror tracing persistence task and paced auditory serial addition task, evaluated participants' tolerance for frustration through task performance and time‐to‐quit measures (Iasonidou et al. 2023; Seymour et al. 2016; Veilleux et al. 2019). Studies also measured mental disorders through mood induction tasks, such as the bubble task, snake task, and speech task, to assess internalizing disorders in children (McGinnis et al. 2021). Multimedia tools, including affective pictures and movie clips, were used to induce targeted emotions like nervousness and fear. For example, Xiang et al. (2024) analyzed pupillary responses to combat scenes to assess resilience, offering nuanced insights into adaptive functioning.

## 4 Discussion

### 4.1 PBA'S Role in Empowering Adolescent Mental Health Assessment

Adolescence is a formative period with substantial physical, cognitive, and socioemotional changes (Patton et al. 2016; Sawyer et al. 2012). These shifts increase vulnerability to emotional fluctuations, self‐concept instability, and social stress, potentially resulting in mental subhealth—a reversible but potentially worsening state if not promptly handled (Dong and Dong 2001; Wu et al. 2021). Reliable assessment during this stage is challenging, as adolescents' self‐awareness is still developing and their self‐reported answers are often influenced by social desirability (De Los Reyes et al. 2015). PBAs offer an alternative by capturing observable mental and behavioral responses in real time rather than relying on introspective judgments. Through performance indicators, PBAs can reveal impairments and mental state changes often missed by self‐reports (Connor and Maeir 2011; Nielsen et al. 2016; Regev and Josman 2020). This sensitivity is particularly valuable for adolescents, who may perform within normal ranges on self‐report scales but exhibit mild mental issues under PBA (Kiss et al. 2024).

In reviewed articles, most CAT‐enhanced PBAs focused on the evaluation of mental disorders, while dimensions such as emotion and sociability, as well as coping and adaptation, have received relatively little attention. This disparity is noteworthy, as these dimensions are crucial to adolescents' psychological development (Knapp 2021; Martinsone et al. 2022; Perzow et al. 2021). Social–emotional and adaptive competencies empower adolescents to manage increasing responsibilities, academic stress, and shifting interpersonal relationships (Collie 2020; Moksnes et al. 2010; Ronen 2021), which are intrinsically dynamic, manifesting through interactions and contextual demands (Folkman 2013; Kuppens and Verduyn 2017). Traditional self‐reports, though useful for assessing internalized and stable traits, are inadequate for capturing these context‐sensitive processes in real time (Anderson et al. 2017). Thus, PBAs that simulate real‐world scenarios and elicit contextual responses may offer a more ecologically grounded means of assessing these competencies (Lievens and De Soete 2012).

PBAs are contextualized, embedding assessment in scenarios that closely mirror adolescents' daily experiences. Such scenario fidelity improves assessment ecological validity by eliciting authentic responses under simulated real‐life stressors or emotionally stimulated scenarios (Stanton and Franz 2015; Fiebig et al. 2020). Beyond static reporting, PBAs are inherently interactive, requiring individuals to respond to evolving demands in continuous scenarios (Andrews‐Todd et al. 2021). These interactive settings foster embodied engagement, enabling the capture of social–emotional and adaptive processes through bodily states like facial expressions, gestures, or physiological arousal (Barsalou 2008; D'Mello et al. 2018). Collectively, these features offer PBAs distinct benefits in assessing adolescents' emotion and sociability, as well as coping and adaptation in ecologically grounded ways.

With growing concerns about social–emotional, adaptive, and other issues of adolescent mental health, the demand for accurate and timely assessment tools has increased (WHO and UNICEF 2024). Future research should focus on designing PBAs for emotion and sociability, as well as coping and adaptation, that correspond with adolescents' developmental characteristics and daily experiences. With familiar scenarios and interactive tasks, such PBAs could accurately measure and timely identify adolescents' social–emotional and adaptive conditions, thus helping them navigate adolescence smoothly and develop healthily.

### 4.2 CAT'S Value and Current Landscape Within Mental Health PBAs

A synthesis of the reviewed articles indicates that CATs have advanced mental health PBAs by transforming the design, capture, and interpretation of psychological evidence. CATs serve four primary functions: tool digitization, scenario creation, data acquisition, and data analysis. These improve the precision, scenario fidelity, evidence complexity, and inferential depth of mental health PBAs, pertaining to different stages of the evidence‐generation process.

Tool digitization and scenario creation are two CAT applications to solve measurement issues of PBA and enhance its design (de Klerk et al. 2016). Tool digitization facilitates precise control of stimulus presentation and response timing, improving PBA's standardization, precision, and reliability (Bauer et al. 2012; de Klerk et al. 2016). It also lowers costs and enables large‐scale, flexible, and efficient assessments, overcoming typical challenges like small sample sizes, uncontrolled conditions, and site dependency (Alfalahi et al. 2022; De Angel et al. 2022; Shute and Rahimi 2017). Scenario creation, particularly when implemented by VR or AR, improves scenario fidelity and ecological validity of PBA, eliciting targeted and authentic responses (Andrews‐Todd et al. 2021; Pieri et al. 2023; Spytska 2024). It should be noted that immersive PBA scenarios require careful design to minimize extraneous processing and maintain coherence, thereby avoiding additional cognitive load (Mayer and Fiorella 2014).

CATs also broaden the scope and depth of psychological evidence within PBAs. CATs facilitate the collection of physiological and behavioral data produced by participants. This capability enables PBAs to be more interactive and embodied, thereby generating more complex and ecologically valid evidence (Barsalou 2008; Niedenthal 2007). The real‐time monitoring of these signals could provide a more detailed picture of underlying mental states (Ates et al. 2024; Bateni and Sigal 2022). After data collection, accurate analysis is essential for deriving meaningful psychological inferences. CATs could combine multimodal data streams, offering complementary insights beyond single modalities (Dai et al. 2023). In mental health PBAs, feature engineering remains the primary analytic paradigm, as it aligns with psychological theory by offering interpretable indicators that connect behavioral features to mental states. Empirical studies demonstrate that ML algorithms outperform traditional linear models in predicting constructs like well‐being and personality (Oparina et al. 2025; Stachl et al. 2020). DL models are relatively less used compared to other fields (Devlin et al. 2019). Although they can automatically learn hierarchical representations for detection, classification, and prediction (LeCun et al. 2015), this often sacrifices interpretability, hence their being known as “black box” models (Ribeiro et al. 2016).

A key challenge in analyzing psychological data with CATs lies in balancing interpretability with predictive precision. Transparent “white‐box” models like linear regression (Weisberg 2005) and decision‐tree algorithms (Safavian and Landgrebe 1991) offer explainable results but lack the predictive power of complex “black‐box” models. Overemphasizing interpretability constrains the psychometric utility of CATs. Some researchers, therefore, argue that prioritizing predictive performance over explanatory power may offer new insights into the dynamics of mental states (Yarkoni and Westfall 2017). In practice, DL approaches have demonstrated promising potential in identifying latent patterns and predicting mental health trajectories. For example, a fine‐tuned BERT model (Devlin et al. 2018) achieved an F1 score of 91.3% in identifying suicide risk on social media (Tanaka and Fukazawa 2024). Furthermore, methodological innovations, such as hidden state analysis in DeBERTa models (Ormerod 2022) and univariate histogram projections for SVMs (Cherkassky and Dhar 2015), exemplify possible pathways to reconcile predictive accuracy with transparency.

CATs also hold promise for delivering personalized and actionable feedback that supports psychological stability and adaptability. Although PBAs move beyond self‐report formats, their results are still expressed in terminology that participants may find difficult to understand. Particularly, adolescents tend to describe mental experiences using everyday language like sadness, loneliness, or anger (JHU and UNICEF 2022), highlighting the need for feedback that resonates with lived experience. Advances in LLMs may address this gap by generating data‐driven, accessible, individualized, and supportive feedback, which may transform PBA from a diagnostic process into a meaningful progress that helps individuals understand and regulate their own mental health.

Collectively, these applications demonstrate the increasing potential of CATs to enhance the applicability, ecological validity, embodiment, and accuracy of mental health PBAs. Future research should focus on developing integrative analysis and feedback frameworks that comprehensively utilize these potentials to improve participants' mental health.

### 4.3 Limitations and Improvements in PBA Scenario Design

The design of scenarios is pivotal to the quality of PBAs for mental health. This is acutely evident in adolescents, who often exhibit mental concerns in some scenarios but not others (De Los Reyes et al. 2015), highlighting the importance of PBA scenario fidelity. Our review finds that current CAT‐enhanced mental health PBAs employ diverse scenarios, including laboratory, social interaction, game, and emotion‐stimulation. Laboratory‐based and social scenarios cover every dimension of mental health, while emotion‐stimulating scenarios primarily target coping and adaptation. VR‐powered game scenarios are particularly common for measuring cognitive functions. Although they can offer safe and controlled conditions for interactions (Riva 2005; Lumsden et al. 2016), these immersive scenarios often introduce excessive stimuli that disrupt attention and impose heavy cognitive loads, reducing measurement precision (Mayer and Fiorella 2014; Neguţ et al. 2016; Lobato‐Camacho et al. 2024). Moreover, overly standardized or culturally neutral scenarios risk reducing participants' emotional resonance (Fang et al. 2022). These limitations reveal the challenge of attaining both high reliability and ecological validity in mental health PBAs (Fletcher and Miciak 2023). Therefore, enhancing the theoretical and cultural relevance, as well as the narrative coherence of scenarios, is essential for improving PBA accuracy and reliability.

Drawing from these challenges, established psychometric instruments, such as self‐report scales, offer valuable insights for PBA scenario design. The scenarios embedded in scale items are typically derived from a rigorous and theory‐driven development process that includes theoretical construction, expert review, and iterative empirical validation (Nunnally and Bernstein 1994; DeVellis and Thorpe 2021). Such procedures ensure that assessment scenarios are theoretically grounded, psychometrically sound, and culturally relevant. For example, emotional ability measures like the Situational Test of Emotional Understanding (MacCann and Roberts 2008) and the Social Emotional Competence Questionnaire (Zhou and Ee 2012) use validated scenarios that simulate interpersonal and emotional challenges in real life. Incorporating such scenarios into PBA design could balance ecological validity with measurement reliability, bridging the methodological rigor of traditional psychometrics with the contextual and interactive advantages of PBAs.

However, scenarios embedded in traditional scales are discrete and static, disrupting narrative flow and immersive engagement. Such fragmentation also heightens participants' cognitive burden, as they need to reconstruct situational meaning (Sweller 2011; Männiste et al. 2019). CATs, especially LLMs, offer promising solutions to these challenges by transforming standardized scale items into storytelling and interactive PBA scenarios (Yang et al. 2024). In doing so, they can enhance interactivity, coherence, and contextual richness while maintaining theoretical grounding. Researchers must carefully manage scenario complexity and participant familiarity while minimizing unnecessary elements (Mayer and Fiorella 2014) to ensure that technologies strengthen the ecological validity, reliability, and contextual fidelity without increasing cognitive load or cultural biases in PBAs.

Future research could use validated psychometric instruments for reference to develop PBA scenarios with stronger theoretical grounding and contextual typicality. CATs can help refine and localize these scenarios to improve storyline coherence and experience fit. Ultimately, such CAT‐enhanced PBAs could enable more effective and engaging assessments of adolescents, facilitating early identification and personalized intervention for mental health issues.

## 5 Conclusion and Limitations

This review systematically mapped the landscape of CAT‐enhanced PBAs for mental health. Through a rigorous multistage screening process across five major databases, we identified 89 articles covering various CAT‐enhanced PBAs that assess mental disorders, cognitive functions, emotion and sociability, coping and adaptation, and self‐cognition. Several critical domains for adolescents are insufficiently explored, like emotion, sociability, coping, and adaptation, which are inappropriately assessed by self‐reports, necessitating developing contextualized and interactive PBAs. CATs strengthen PBAs through data analysis, physiological and behavioral data collection, scenario creation, and tool digitization, enhancing PBA design, evidence diversity, and analytical ability. However, their potential for generating predictive analytics and using LLMs for PBA feedback remains untapped. PBA scenarios are diverse, spanning laboratory, social interaction, game, and emotion‐stimulating contexts. To mitigate potential issues such as poor reliability, excessive cognitive burden, and insufficient typicality in PBA, LLMs can be employed to adapt validated scales and generate coherent, ecologically valid, and theoretically grounded PBA scenarios. With CAT‐enhanced PBAs, improve the effectiveness and accuracy in identifying mental health issues, thus designing more targeted intervention strategies to foster the growth and development of adolescents.

This review underscores the value of CAT‐enhanced PBAs in addressing the limitations of traditional assessment methods, offering a framework for integrating specific technologies into diverse mental health dimensions. Expanding CAT applications and incorporating context‐sensitive scenarios could enhance early identification of mental health concerns, fostering evaluations that support psychological stability and adaptability. Meanwhile, this review reveals an important gap in the literature regarding the limited research focused on teenagers. Future research should adapt validated computer‐assisted PBA paradigms developed for adults to adolescents, refining scenario, task, and feedback designs to suit developmental traits.

Despite these contributions, this review has limitations. First, it overlooks the role of group‐level psychological constructs, such as collective emotion, cohesion, and shared beliefs, which are essential for understanding societal mental health outcomes (Abrutyn 2023; Goldenberg 2024; McIntyre et al. 2018). Future research should adopt multilevel frameworks that integrate individual and collective variables to inform interventions addressing personal and community mental health. Second, this review synthesizes evidence descriptively due to the methodological heterogeneity of included studies, without performing a risk‐of‐bias evaluation or a quantitative evaluation of PBA efficacy and reliability. As such, the findings should be interpreted as a mapping of the field rather than an appraisal of evidence strength. Future meta‐analyses are needed to generate statistically robust insights into PBA efficacy, identify methodological strengths and weaknesses, and guide the development of more reliable and effective mental health PBAs (Gurevitch et al. 2018). Finally, although we did not pre‐register a review protocol, we sought to maintain methodological rigor by adhering to the PRISMA‐ScR reporting guidelines and providing full details of the search strategy, screening, and data extraction. This approach enhances transparency and allows readers to appraise the review process and reproducibility.

## Funding

This work was supported by National Key Research and Development Program of China (2021YFC3340800).

## Conflicts of Interest

The authors declare no conflicts of interest.

## Data Availability

Data sharing not applicable to this article as no datasets were generated or analysed during the current study.

## Appendix A The Detailed Search Strings Used in Each Database Are Presented Below

**Table:**

Database	Fields searched	Search string
Web of Science	Topic (includes Title, Abstract, Author Keywords, Keywords Plus)	(TS = well‐being OR TS = mental health OR TS = psychology OR TS = mental disorder) AND (TS = assessment OR TS = measure OR TS = test) AND (TS = computerized OR TS = computer assisted OR TS = artificial intelligence OR TS = machine learning) and 2025 or 2024 or 2023 or 2022 or 2021 or 2020 or 2019 or 2018 or 2017 or 2016 or 2015 (Publication Years) and English (Languages) and Computer Science or Psychology or Psychiatry or Education Educational Research (Research Areas) and Article or Meeting or Early Access or Book (Document Types)
EI Engineering Village	Classification, Language, Document Type, Year	(((((((((well‐being OR mental health OR psychology OR mental disorder) AND (assessment OR measure OR test) AND (computerized OR computer assisted OR artificial intelligence OR machine learning))) AND (({ca} OR {ja} OR {cp} OR {ch}) WN DT)) AND ({english} WN LA)) AND (({723.4} OR {723} OR {971} OR {901.2}) WN CL)))) AND ((2025 OR 2024 OR 2023 OR 2022 OR 2021 OR 2020 OR 2019 OR 2018 OR 2017 OR 2016 OR 2015) WN YR))
Scopus	TITLE‐ABS‐KEY (Title, Abstract, Keywords)	TITLE‐ABS‐KEY (“mental health” OR “well‐being” OR “psychology” OR “mental disorder”) AND TITLE‐ABS‐KEY (“assessment” OR “measure” OR “test”) AND TITLE‐ABS‐KEY (“computerized” OR “computer assisted” OR “artificial intelligence” OR “machine learning”) AND PUBYEAR >2015 AND PUBYEAR <2025 AND (LIMIT‐TO (DOCTYPE, “ar”) OR LIMIT‐TO (DOCTYPE, “cp”) OR LIMIT‐TO (DOCTYPE, “ch”)) AND (LIMIT‐TO (LANGUAGE, “English”)) AND (LIMIT‐TO (SUBJAREA, “PSYC”) OR LIMIT‐TO (SUBJAREA, “COMP”) OR LIMIT‐TO (SUBJAREA, “SOCI”))
ProQuest‐APA PsycINFO	Subject Heading, Title, Abstract, Document Type, Language, Publication Date	(((MH “Well Being”) OR (MH “Mental Health”) OR TI(“well‐being” OR “mental health” OR “mental disorder”) OR AB(“well‐being” OR “mental health” OR “mental disorder”)) AND ((MH “Psychological Assessment”) OR (MH “Measurement”) OR TI(assessment OR measure* OR test) OR AB(assessment OR measure* OR test)) AND (TI(computerized OR “computer‐assisted” OR “artificial intelligence” OR “machine learning”) OR AB(computerized OR “computer‐assisted” OR “artificial intelligence” OR “machine learning”))) AND rtype.exact(“Peer Reviewed Journal” OR “Chapter” OR “Journal Article” OR “Conference Proceedings” OR “Book” OR “Journal”) AND la.exact(“English”) AND pd.(20150101–20251231)
PubMed	MeSH Terms, Title/Abstract, Publication Date, Language, Publication Type	((“Mental Health”[Mesh] OR “Mental Disorders”[Mesh] OR “Psychology”[Mesh] OR “well‐being”[tiab] OR “mental health”[tiab] OR “psychology”[tiab] OR “mental disorder*”[tiab]) AND (“Psychological Tests”[Mesh] OR “Psychometrics”[Mesh] OR “Diagnostic Techniques and Procedures”[Mesh] OR assessment[tiab] OR measure*[tiab] OR test[tiab]) AND (“Artificial Intelligence”[Mesh] OR “Machine Learning”[Mesh] OR “Computers”[Mesh] OR computerized[tiab] OR “computer assisted”[tiab] OR “artificial intelligence”[tiab] OR “machine learning”[tiab] OR “AI”[tiab])) AND (“2015/01/01”[Date ‐ Publication]: “2025/12/31”[Date ‐ Publication]) AND English[la] Filters: Books and Documents, Clinical Conference, Clinical Study, Clinical Trial, Comparative Study, Congress, Controlled Clinical Trial, Evaluation Study, Observational Study, Randomized Controlled Trial, Twin Study, Validation Study, English

## Appendix B

**Table:**

First Author (Date)	Sample age group	Mental health components	CAT application	PBA scenario
Settanni and Marengo (2015)	Adults	Mental disorders; Coping and adaptation	Data analysis	Social interaction
Campbell et al. (2015)	Adolescents	Cognitive functions; Emotion and sociability	Digital tool	Laboratory‐based
Nowakowski et al. (2015)	Adults	Emotion and sociability	Data acquisition	Social interaction
Seymour et al. (2016)	Adolescents	Coping and adaptation	Digital tool; Scenario creation	Emotion‐stimulating
Fitts et al. (2016)	Adults	Emotion and sociability	Digital tool	Laboratory‐based
Parsons and Carlew (2016)	Adults	Mental disorders	Digital tool; Scenario creation; Data acquisition	Laboratory‐based
Wu et al. (2016)	Adults	Mental disorders	Digital tool; Scenario creation; Data analysis	Laboratory‐based
Lenehan et al. (2016)	Adults	Cognitive functions	Digital tool; Scenario creation	Laboratory‐based
Pile et al. (2017)	Adolescents	Emotion and sociability	Digital tool; Scenario creation	Social interaction
Neguț et al. (2017)	Adolescents	Mental disorders	Digital tool; Scenario creation	Laboratory‐based
Öztoprak et al. (2017)	Adolescents	Mental disorders	Digital tool; Data acquisition; Data analysis	Laboratory‐based
Broch‐Due et al. (2018)	Adults	Emotion and sociability	Digital tool; Data acquisition	Emotion‐stimulating
Nguyen et al. (2018)	Adults	Mental disorders	Data analysis	Social interaction
Compton et al. (2018)	Adults	Mental disorders	Data analysis	Social interaction
Schoch‐Ruppen et al. (2018)	Adults	Mental disorders; Coping and adaptation	Data analysis	Social interaction
Islam et al. (2018)	Adults	Mental disorders	Data analysis	Social interaction
Lin et al. (2018)	Adults	Mental disorders	Digital tool; Scenario creation	Laboratory‐based
McArthur et al. (2019)	Adolescents	Self‐cognition	Digital tool	Laboratory‐based
Leontyev et al. (2019)	Adults	Mental disorders	Digital tool; Data analysis	Laboratory‐based
Jing et al. (2019)	Adults	Mental disorders	Data analysis	Laboratory‐based; Social interaction
Yang et al. (2019)	Adults	Mental disorders	Data analysis	Social interaction
Poznyak et al. (2019)	Adolescents	Emotion and sociability	Digital tool; Scenario creation	Laboratory‐based
Chandra Guntuku et al. (2019)	Adults	Coping and adaptation	Data analysis	Social interaction
Myruski et al. (2019)	Adults	Emotion and sociability	Digital tool; Scenario creation	Laboratory‐based
Veilleux et al. (2019)	Adults	Coping and adaptation	Digital tool; Scenario creation	Emotion‐stimulating
König et al. (2019)	Adults	Emotion and sociability	Data analysis	Social interaction
Huang et al. (2019)	Adults	Cognitive functions	Digital tool	Laboratory‐based; Game
Zhao et al. (2019)	Adults	Mental disorders	Data analysis	Laboratory‐based
Hemtanon et al. (2020)	Adults	Mental disorders	Data analysis	Social interaction
Muszynski et al. (2020)	Adolescents	Mental disorders	Data analysis	Social interaction
Birnbaum et al. (2020)	Mixed age	Mental disorders	Data analysis	Social interaction
Purwandari et al. (2020)	Adults	Mental disorders; Coping and adaptation	Data analysis	Social interaction
Roy et al. (2020)	Adults	Mental disorders	Data analysis	Social interaction
Sivak and Smirnov (2020)	Adolescents	Mental disorders; Emotion and sociability	Data analysis	Social interaction
Wangkawan et al. (2020)	Adolescents	Cognitive functions	Digital tool	Laboratory‐based
Marín‐Morales et al. (2021)	Adults	Cognitive functions	Digital tool; Scenario creation; Data analysis	Laboratory‐based; Game
Farhoumandi et al. (2021)	Adults	Emotion and sociability	Digital tool; Scenario creation; Data analysis	Laboratory‐based
Tsai et al. (2021)	Adults	Cognitive functions	Digital tool; Scenario creation; Data analysis	Game
Lu et al. (2021)	Adults	Mental disorders	Digital tool	Laboratory‐based
McGinnis et al. (2021)	Adolescents	Mental disorders	Data acquisition	Emotion‐stimulating
Corsi et al. (2021)	Mixed age	Emotion and sociability	Digital tool; Scenario creation	Laboratory‐based
Kim et al. (2022)	Adults	Cognitive functions; Coping and adaptation	Digital tool; Scenario creation; Data acquisition	Game
Stark et al. (2022)	Adults	Mental disorders	Data acquisition; Data analysis	Laboratory‐based
Deb et al. (2022)	Adults	Mental disorders	Data analysis	Social interaction
Kelley et al. (2022)	Adults	Mental disorders; Emotion and sociability	Data analysis	Social interaction
Sheoran and Srivastava (2022)	Adults	Mental disorders	Data analysis	Laboratory‐based
Ali et al. (2022)	Adults	Mental disorders; Self‐cognition	Data analysis	Social interaction
Walia et al. (2022)	Adults	Mental disorders	Digital tool; Scenario creation	Social interaction
Singh and Singh (2022)	Mixed age	Mental disorders; Coping and adaptation	Data analysis	Social interaction
Liu, Zhong, et al. (2022) and Liu, Lu, et al. (2022)	Adults	Mental disorders; Emotion and sociability; Self‐cognition	Data analysis	Social interaction
Llanes and Reyes (2022)	Adults	Coping and adaptation	Data analysis	Laboratory‐based
Liu, Zhong, et al. (2022) and Liu, Lu, et al. (2022)	Mixed age	Mental disorders	Data analysis	Social interaction
Kjærstad et al. (2022)	Adults	Emotion and sociability	Digital tool; Scenario creation	Social interaction
Jyotsna et al. (2023)	Adults	Coping and adaptation	Data acquisition; Data analysis	Emotion‐stimulating
Danousis and Goumopoulos (2023)	Adults	Cognitive functions	Digital tool; Scenario creation; Data acquisition; Data analysis	Game
Ibrahim et al. (2023)	Adults	Cognitive functions	Digital tool; Scenario creation	Game
Iasonidou et al. (2023)	Adults	Coping and adaptation	Digital tool; Scenario creation	Emotion‐stimulating
Ortuño‐Miró et al. (2023)	Adolescents	Mental disorders	Digital tool; Data acquisition; Data analysis	Laboratory‐based
Bruno et al. (2023)	Adults	Mental disorders	Data analysis	Laboratory‐based
Voleti et al. (2023)	Adults	Mental disorders	Data analysis	Social interaction
Villa‐Pérez et al. (2023)	Adults	Mental disorders	Data analysis	Social interaction
Huang et al. (2023)	Adults	Mental disorders	Data analysis	Laboratory‐based
Badr et al. (2023)	Adults	Coping and adaptation	Digital tool; Data analysis	Laboratory‐based
Oesten et al. (2023)	Adults	Coping and adaptation	Data analysis	Social interaction
Wright‐Berryman et al. (2023)	Adults	Mental disorders	Data analysis	Social interaction
Xiang et al. (2024)	Adults	Coping and adaptation	Data acquisition; Data analysis	Emotion‐stimulating
Harshit et al. (2024)	Adults	Emotion and sociability	Data analysis	Social interaction
Yadawad et al. (2024)	Adults	Mental disorders	Digital tool; Data acquisition; Data analysis	Laboratory‐based; Social interaction; Emotion‐stimulating
Luo et al. (2024)	Adolescents	Mental disorders	Data analysis	Laboratory‐based
Jiang et al. (2024)	Adults	Mental disorders	Data analysis	Social interaction
Fantozzi et al. (2024)	Adolescents	Emotion and sociability	Digital tool; Scenario creation; Data acquisition	Social interaction
Rajendran et al. (2024)	Adults	Coping and adaptation	Data acquisition; Data analysis	Laboratory‐based
Finze et al. (2024)	Adults	Emotion and sociability; Coping and adaptation; Self‐cognition	Data analysis	Social interaction
Zeulner et al. (2024)	Adults	Emotion and sociability; Coping and adaptation	Data analysis	Social interaction
Rochotte et al. (2024)	Adults	Mental disorders	Data analysis	Social interaction
Shin et al. (2024)	Adults	Mental disorders	Data analysis	Social interaction
Andrikopoulos et al. (2024)	Adults	Mental disorders	Digital tool; Data acquisition; Data analysis	Laboratory‐based
Tezer et al. (2025)	Adolescents	Cognitive function	Digital tool	Laboratory‐based
Raina (2025)	Adults	Emotion and sociability	Data acquisition; Data analysis	Emotion‐stimulating
Mutersbaugh et al. (2025)	Adolescents	Mental disorders	Data acquisition; Data analysis	Laboratory‐based
Hoffmeister et al. (2025)	Adults	Cognitive function	Digital tool	Laboratory‐based
Ding et al. (2025)	Mixed age	Mental disorders	Data analysis	Laboratory‐based; Emotion‐stimulating
DesRuisseaux et al. (2025)	Adults	Cognitive function	Digital tool	Laboratory‐based
Pless et al. (2025)	Adults	Cognitive function	Digital tool; Scenario creation	Game
Chen et al. (2025)	Adults	Mental disorders	Data analysis	Laboratory‐based
Stone et al. (2025)	Adults	Mental disorders	Digital tool; Data acquisition; Data analysis	Laboratory‐based
Mejía et al. (2025)	Adolescents	Cognitive function	Digital tool; Scenario creation	Game
Kho et al. (2025)	Mixed age	Mental disorders	Data analysis	Social interaction
Olah et al. (2025)	Adults	Mental disorders	Data analysis	Laboratory‐based
